# Idiopathic Megacolon—Short Review

**DOI:** 10.3390/diagnostics11112112

**Published:** 2021-11-15

**Authors:** Adrian Constantin, Florin Achim, Dan Spinu, Bogdan Socea, Dragos Predescu

**Affiliations:** 1 General and Esophageal Clinic, Sf. Maria Clinical Hospital Bucharest, Carol Davila University of Medicine and Pharmacy, 011172 Bucharest, Romania; dradiconstantin@yahoo.com (A.C.); florin.achim@umfcd.ro (F.A.); 2 Department of Urology, Central Military Emergency University Hospital Bucharest, Carol Davila University of Medicine and Pharmacy, 010825 Bucharest, Romania; dan.spinu@yahoo.co.uk; 3 Department of Surgery, Sf. Pantelimon Emergency Clinical Hospital Bucharest, Carol Davila University of Medicine and Pharmacy, 021659 Bucharest, Romania; bogdan.socea@umfcd.ro

**Keywords:** idiopathic megacolon, etiopathogenic theories of idiopathic megacolon, surgical treatment

## Abstract

Introduction: Idiopathic megacolon (IM) is a rare condition with a more or less known etiology, which involves management challenges, especially therapeutic, and both gastroenterology and surgery services. With insufficiently drawn out protocols, but with occasionally formidable complications, the condition management can be difficult for any general surgery team, either as a failure of drug therapy (in the context of a known case, initially managed by a gastroenterologist) or as a surgical emergency (in which the diagnostic surprise leads additional difficulties to the tactical decision), when the speed imposed by the severity of the case can lead to inadequate strategies, with possibly critical consequences. Method: With such a motivation, and having available experience limited by the small number of cases (described by all medical teams concerned with this pathology), the revision of the literature with the update of management landmarks from the surgical perspective of the pathology appears as justified by this article. Results: If the diagnosis of megacolon is made relatively easily by imaging the colorectal dilation (which is associated with initial and/or consecutive clinical aspects), the establishing of the diagnosis of idiopathic megacolon is based in practice almost exclusively on a principle of exclusion, and after evaluating the absence of some known causes that can lead to the occurrence of these anatomic and clinical changes, mimetically, clinically, and paraclinically, with IM (intramural aganglionosis, distal obstructions, intoxications, etc.). If the etiopathogenic theories, based on an increase in the performance of the arsenal of investigations of the disease, have registered a continuous improvement and an increase of objectivity, unfortunately, the curative surgical treatment options still revolve around the same resection techniques. Moreover, the possibility of developing a form of etiopathogenic treatment seems as remote as ever.

## 1. Introduction

Idiopathic megacolon is a condition characterized by an enlarged colon and aperistaltic syndrome in the absence of a detectable cause. The main symptom is considered chronic constipation, refractory to drug treatment and without surgical indication [1,2]. IM affects both sexes and the symptoms develop early in childhood or in adulthood [3]. The condition has a relatively unknown etiopathogenesis, but according to the nosological framework, the term idiopathic megacolon primarily excludes congenital nerve plexus disorders (Hirschsprung’s disease), or colonic changes secondary to a systemic disorder (so-called “colonic pseudo obstruction”). The terminology used in the literature for this pathology can lead to confusion by the name of either “idiopathic colon” or “acquired megacolon” depending on the authors, but referring to the same pathological entity.

The incidence of the disease is not quantified in the literature. The authors [2,3] address this aspect in vague terms, taking as a diagnostic benchmark the onset of symptoms (which triggers the investigational management and finally the issuance of the diagnosis). It is observed that the condition affects both sexes and the clinical onset may be in childhood or adulthood, without being able to extract additional data, for objective reasons (reduced number of cases, a delay in diagnosis in close connection with symptoms that are also related the patient’s tolerance threshold).

The lack of an etiopathogenic therapy forced the addressability of the cases to the surgeon, with the colon resection techniques confirming their effectiveness over time in IM. If the changes specific to the disease do not affect the rectum, a simple colectomy appears as the ideal option. The physiopathological interest of the rectum makes it necessary to associate it in surgical resection, with procto-colectomy being defined as the optimal technique [2]. However, the effectiveness of therapeutic approaches to patients with idiopathic megacolon cannot be rigorously quantified, hence the lack of clear therapeutic protocols. In the literature, most studies in this direction which include a small number of patients (3–50) are retrospective with a short period of post-therapeutic monitoring (0.5–7 years) [4]. Consequently, the interest from a surgical perspective for this condition is easy to understand. The experience of our clinic confirms statistical data of other authors. Thus, in the period 2000–2020, we hospitalized in clinic a number of nine patients, all with severe intestinal transit disorders, under the appearance of a sub-occlusive/occlusive syndrome. All patients were operated on in a delayed emergency, without the certainty of a firm diagnosis of IM, benefiting from a more or less extensive resection in accordance with the extent of the lesion.

As extent literature reveals, there is an inconsistency in the approach to the subject, with many ambiguities, starting from the terminology (frequent confusions between idiopathic megacolon, Hirschsprung’s disease, etc.). In addition, we found a fragmentation in the approach to the subject either in the form of simple case presentations or on niche topics dealing with isolated aspects of the disease (genetics, HP and IHC, imaging, etc.). As a consequence, it becomes difficult to create a unitary overview accessible to the clinician. With such a motivation, and having at his disposal experience limited by the small number of cases (described by all medical teams concerned with this pathology), the revision of the literature with the update of management guidelines from the surgical perspective of the pathology appears as justified by this article.

## 2. Clinical Manifestations

The anamnesis of the patients must be a rigorous one, which should carefully investigate the eating habits (eating hours, type of food, etc.), the dynamics of digestive transit, the administration of drugs (e.g., antidepressants, laxatives, nonsteroidal anti-inflammatory drugs, etc.), daily physical activity, and elements of mental impairment (stress, depression, endocrine syndromes such as hypothyroidism, etc.). The usefulness of the anamnestic evaluation is obvious in the diagnosis of MI in order to exclude other mimetic pathologies, which associate severe constipation as an essential element from a clinical point of view (Table 1). Physical examination has its role, often identifying abdominal distension, alternate dull meteorism, attenuation, or even the disappearance of intestinal sounds, with the identification of abdominal masses, often large feces. Rectal exam involves the correct assessment of sphincter tonicity, detection of intraluminal masses (fecalomas), and parietal changes. The symptoms of the acquired megacolon differ from those of the congenital megacolon. In IM, the adult is the target age category, with constipation, abdominal pain, gas distension, and abdominal discomfort as characteristically present. Although it may overlap with the clinical picture of patients with irritable bowel syndrome [5,6], it is not known exactly what percentage of patients with irritable bowel disease may have concomitant IM. On the other hand, children with megacolon generally have meconium ileus in the neonatal period (usually in association with Hirschsprung’s disease), possibly occlusive/sub-occlusive syndromes in childhood. Later, as a characteristic, the formation of fecalomas is described [7].

Special mention should be made of Ogilvie syndrome. In the literature, we have not encountered its classification as a subcategory of the idiopathic megacolon. In fact, we subscribe to this attitude. Although there are a number of common aspects (clinical picture, absence of an etiopathogenic morphological substrate in the colon, anatomoclinical changes of the colon), certain aspects significantly differentiate the two pathological entities. In the case of Ogilvie syndrome, the correlation with a trigger (severe trauma, major surgery, and certain drug treatments) defines this condition as “secondary”, disqualifying it for inclusion in the nosological framework of the idiopathic megacolon.

## 3. Etiopathogenic Theories

The pathogenesis of the disease is still controversial, although a number of hypotheses are already documented. The highlighting of some HP and IHC changes on the resection specimens compared to the healthy control population, showed a rather varied lesional heterogeneity, making it difficult to invoke, in terms of etiopathogenic importance, the greater or lesser responsibility of any of the identified factors. Associated or not, the decreased cellularity of nerve plexuses [8,9], alterations of extramural spinal innervation [10], neuromuscular disorders [11], and decreased number of Cajal cells [12], are not described as possible causes. Unfortunately, one thing seems very clear to us: immunohistochemical studies (IHC) are not consistent in supporting any of these hypotheses [13,14], discovering new and possible changes as an etiopathogenic source. Attentively, we understand that there is a damage at the parietal colonic level, but who generates it, what mechanisms determine these changes, and what is the target population group, if there is one, are questions to which we do not have a clear answer but only speculations. A good example is the invocation of events and lesions described in children with aperistaltic megacolon, in which the absence of tendon membranes from the myenteric plexus is detected as well as alterations (quasi-complete atrophy) of the tendon fiber network in the muscle [15,16,17]. These lesions were later described in adults, thus leaving open the possibility of detection and other changes related to the causality of the presence of the megacolon.

### 3.1. Cajal Cells

Changes in Cajal interstitial cell numbers are described in patients with idiopathic megacolon or chronic constipation by numerous studies and suggested as an etiopathogenic basis [18,19,20,21,22,23,24]. They are considered a true intestinal pacemaker (“little brain” at the enteral level); however, starting from the premise that their number decreases physiologically with age anyway and by the fact that a number of other communications do not confirm their involvement in the etiopathogenesis of idiopathic megacolon, this theory remains for the moment only a new subject of controversy [8].

### 3.2. Smooth Muscle Cells

Another theory suggests that the development of the idiopathic megacolon is based on degenerative changes in smooth muscle tissue [20,25]. The alteration of colonic muscle cells in the case of idiopathic megacolon in cats [26,27], and similar changes identified in mice [28], are also documented. In corollary, a 2006 study in patients with idiopathic megacolon showed a reduction in colonic smooth cell myofilaments and an altered expression of the pattern of myosin-type muscle markers with heavy chain or histone deacetylase 8, despite the normal histological appearance of standard hematoxylin-eosin staining. [29].

### 3.3. Intramural Tendon Fiber Network

It is considered that three elements are essential for normal motility: smooth muscles, the intramural connective tendon network, and the integrity of the myenteric plexuses [17]. In IM, atrophy of the tendon network causes the disappearance of peristalsis completely [9] and allows uncontrolled dilation of the colon [30]. The functional consequences of the atrophy of the tendon structures at the level of its own muscle become obvious if we consider the investigations carried out by Rollo et al. [31], who showed that peristalsis is entirely dependent on the integrity of this network. During the contraction of the longitudinal muscle fibers, the tendon network modulates the dilation of the layer of uncontracted circular muscles. At the same time, the tendon network does not allow the elongation of the relaxed longitudinal fibers during the contraction of the circular muscular layer. Both phenomena that alternate in the colonic motor activity are thus dependent on the integrity of the tendon network, being coordinated by the enteric nerve plexuses and determined by the propulsion of the colonic content [8].

It appears extremely interesting that, in IM, aperistaltic syndrome is not accompanied by colonic wall hypertrophy. Moreover, it even describes colonic parietal atrophy in cases of idiopathic megacolon, especially in the longitudinal muscles.

On the other hand, it is known that both smooth muscle cells and collagen elements have a mesenchymal origin. This explains why smooth muscle cells synthesize type I and III collagen [32,33,34,35,36,37]. *Growth factor beta-1* induces collagen synthesis in smooth muscle cell cultures [37]. This supports the hypothesis of a defect in the synthesis of collagen in smooth, genetically determined muscles, which underlies the appearance of idiopathic megacolon, especially in young individuals. In support of this hypothesis, a colonic perforation is reported in a patient with a form of Ehlers-Danlos syndrome with a significant defect in type III collagen synthesis [31]. Colon dilatation phenomena have also been described in patients with other pathologies related to altered connective tissue metabolism, such as scleroderma or amyloidosis [38,39,40,41].

Moreover, the network of tendon fibers in the muscles is particularly rich in type III collagen fibers. It has a relatively high metabolism of hydroxyproline compared to type I collagen. As an important consequence, the production of type III collagen is affected in ascorbate deficiency, as documented in tissue culture experiments [33,34].

### 3.4. Pelvic-Perineal Muscle Disorders

Some studies show pelvic-perineal muscle disorders of up to 40% in patients with megacolon. Controversy arises as to whether these disorders are part of the initial systemic neuromuscular disorders, a consequence of colonic atony, or a cause of colonic distension. Rectal distension, as found in pelvic-perineal muscle disorders, inhibits colonic tonicity through a negative feedback mechanism mediated by a viscero-visceral reflex [42]. Patients with chronic constipation, due to pelvic-perineal muscle disorders, also have an inadequate postprandial colonic motor response [43]. The aspects are with direct therapeutic involvement. In the absence of a correction of these pelvic-perineal dysfunctions, a subtotal colectomy with ileorectal anastomosis will not improve the symptoms related to the slowed digestive transit [44,45,46].

### 3.5. Genetic Appearance

Many of the functional and morphological changes in colonic smooth muscle tissue, interstitial structures, and nerve structures, were detected in mice during the overexpression of the Hoxa-4 gene [47]. It encodes a specific transcription factor that modulates cell positional identity [48]. In mice with an overexpressed Hoxa-4 gene, a short segment with aganglionosis in the terminal colon was detected. Moreover, the lymph nodes present in the longitudinal muscles were malpositioned. In individuals with significant impairment, death occurs early postnatal. In the case of a less severe impairment, survival to adulthood may be recorded. These data suggest that the idiopathic megacolon may be caused by genetic changes involving the distribution and interaction of different cellular components in the colonic wall. At the same time, the involvement of extrinsic factors (diet, pharmacological substances) can be considered [49] as associated mechanisms.

## 4. Diagnostic Criteria for the Acquired Megacolon

**Figure 1:**
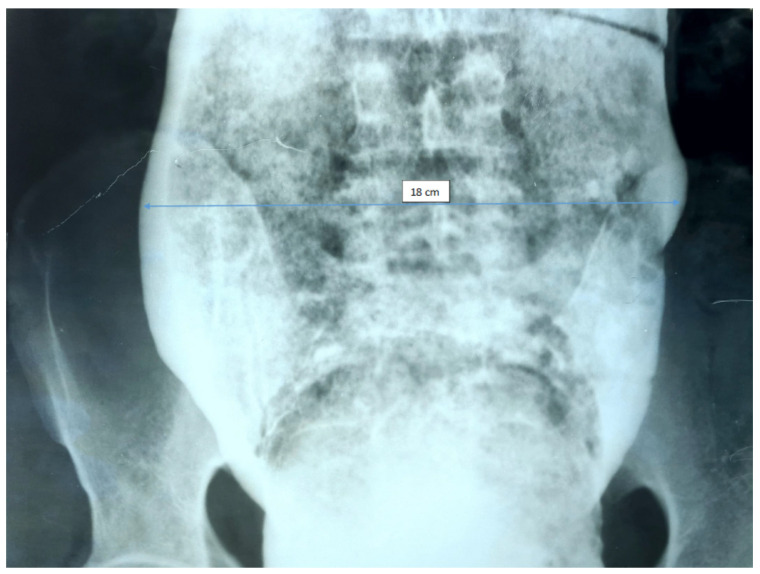
Irigographic examination—rectal evaluation sequence. Impressive dimensions of the rectum are found—about 18 cm diameter lumen, full of fecal matter, occupying the entire pelvis and compressing the adjacent viscera.

**Figure 2:**
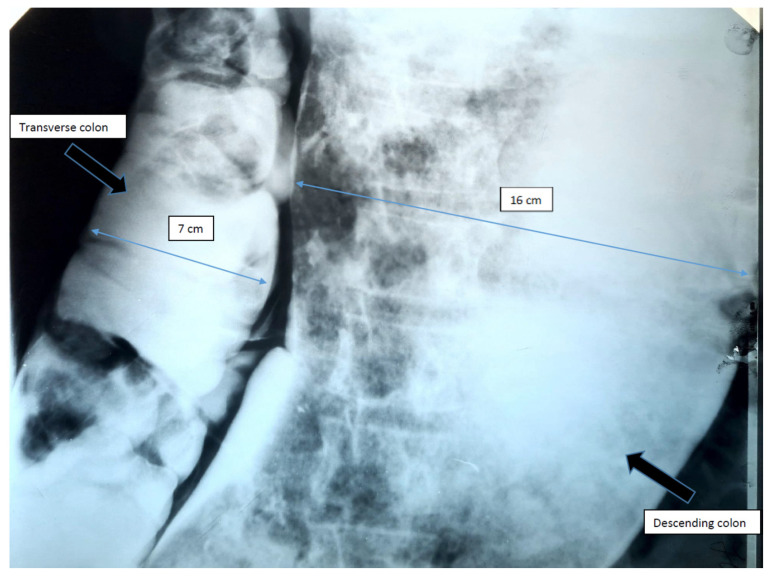
Irigographic examination—sequence. Monstrous segmental dilation located at the level of the descending colon and sigmoid is identified, the dimensional evaluation showing a lumen diameter of approximately 16 cm. It is noticeable the erasure of the haustrations and the envelopes of the colon, as well as numerous remains, organized in multiple faeces. Transverse colon slightly dilated, but with a caliber relatively within normal limits.

**Figure 3:**
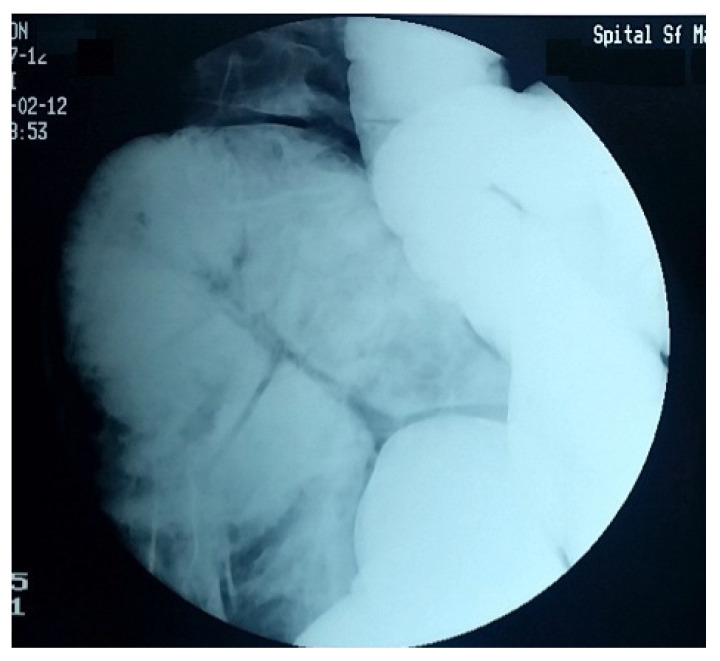
Colon with impressive length, with multiple volutes and important dilation.

**Figure 4:**
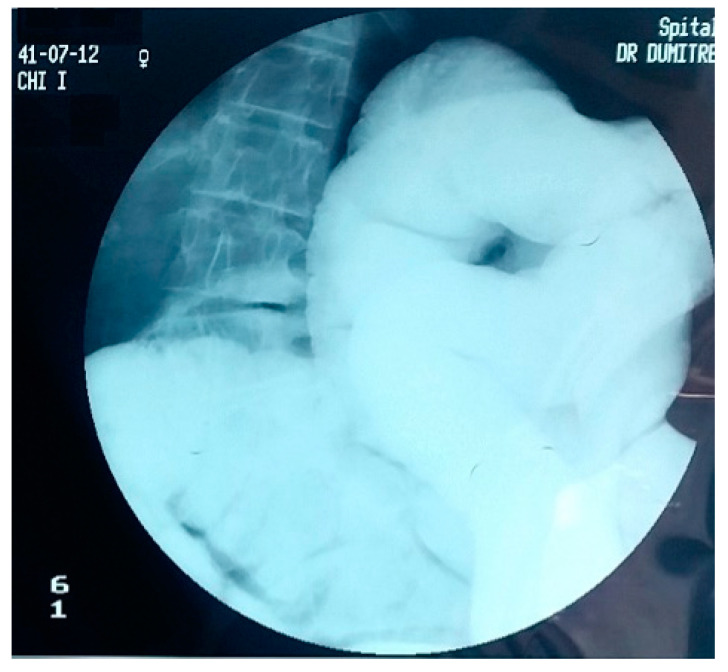
Barium enema. Colon with the deletion of the haustrations at the level of the descendant and the sigmoid.

**Figure 5:**
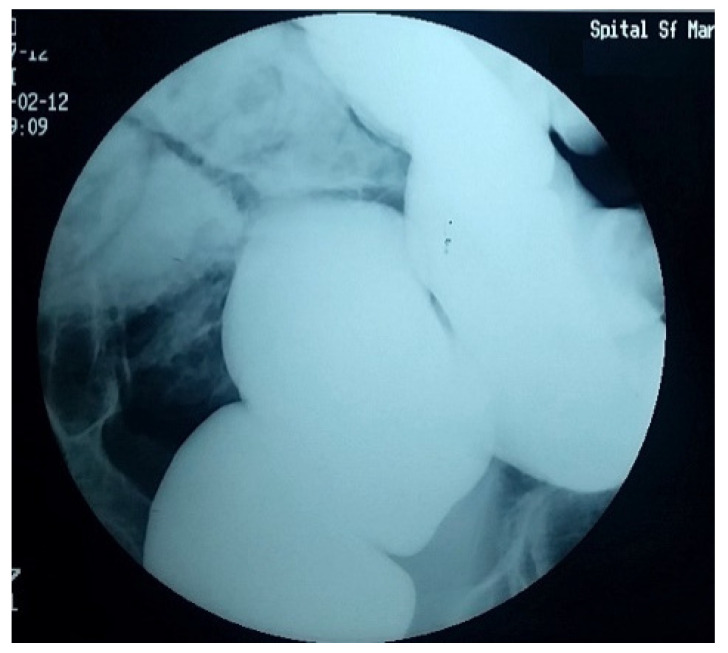
Significant rectal dilation.

**Figure 6:**
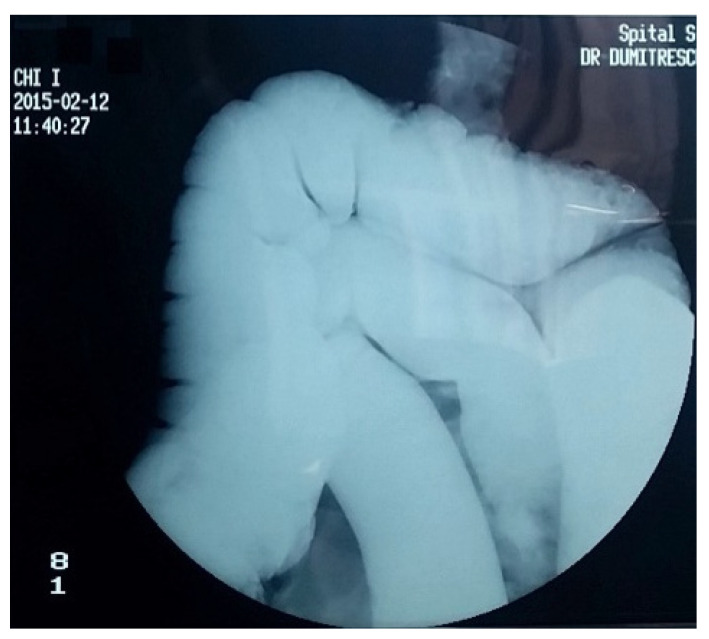
Significant reflux in the terminal ileum, which appears dilated, the consequence of transit disorders and fecal stasis in the colon.

**Figure 7:**
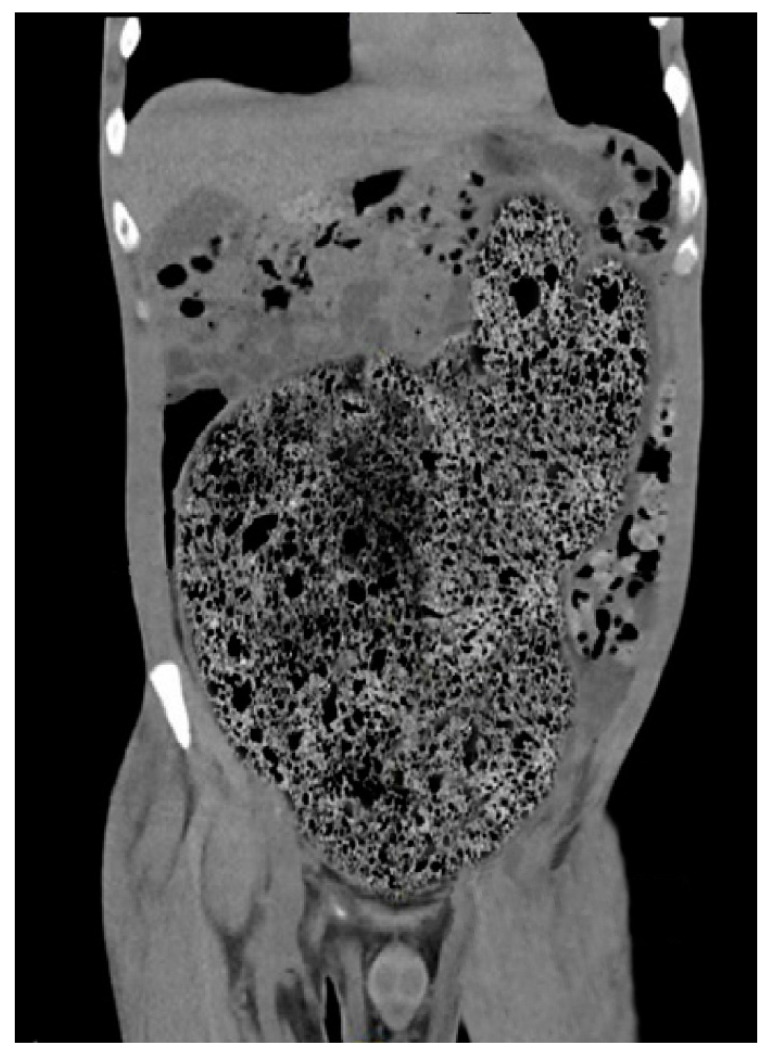
CT scan with idiopathic megarectum and megacolon.

Over the years, various diagnostic criteria for megacolon have been used. In 1985, studies of the colon using double contrast barium enema established normal diameter of the recto-sigmoid below 6.5 cm at the pelvis. Apparently, the diagnosis of IM is an easy one, the main criterion being the imaging one, by the dimensional appreciation evaluated by the diameter of the loop, namely over 6.5 cm at the level of the pelvic loop [50,51], over 8 cm at the level of the ascending colon, and over 12 cm at the cecum. However, the rigor of these benchmarks has never been so unanimous or accepted. Thus, the limit of 10 cm for the diagnosis of megacolon was established with variations of 2–3.5 cm depending on the studies (Figure 1 and Figure 2). Other studies have proposed as a diagnostic criterion the identification of the sigmoid loop above the iliac crest on barium enema or the persistence of symptoms after segmental colectomy. Studies have shown that radiological examinations used for diagnosis show significant variability and the location of measurements is often insufficiently specified (Figure 3, Figure 4, Figure 5 and Figure 6). Moreover, using only sigmoid measurements, one can neglect a group of patients who have isolated dilatation strictly in the proximal colon [52]. Abdominal and pelvic computed tomography provide much more accurate information in this direction (Figure 7). In addition, CT has the ability to assess colorectal parietal thickness, edema, and inflammatory hyperemia. A possible diagnostic solution, which allows a detailed exploration, with a good dimensional evaluation of the colon in its various segments is virtual colonoscopy.

The diagnostic criteria of MI based on imaging studies, as well as on a specific symptomatology (abdominal pain, constipation, and abdominal gas distension), are not sufficient to differentiate MI from other pathological entities.

The exclusion of organic, mimetic diseases as a symptomatology and imaging aspect, is an important step in establishing the diagnosis. Additional techniques can be used. Colonoscopic examination, which appears as a logical indication, especially for biopsy collection, is actually difficult to perform; colon preparation is often impossible or inadequate and biopsy specimens do not turn out as expected. The HP aspects in the literature are not the result of endoscopic biopsies but of resection pieces. In addition, the risk of perforation during exploration is enormous.

Some communications recommend in doubtful cases of IM, or when imaging details are equivocal, to resort to colonic motility tests, such as the use of barostat-controlled colonic-controlled 10-cm-long infinitely compliant balloon.

Moreover, the association with the administration of colic prokinetics (neostigmine) can guide the therapeutic decision by selecting patients as candidates for oral treatment with anticholinesterases, such as pyridostigmine [38].

Investigating the motor activity of the colon can highlight the increase of the resting tone at the level of the anal sphincter, an increased incidence of peristaltic waves with low pressure, and diminished rectal reflex at the same time as the increase of the rectal capacity. The consequence is the presence of the megarectum or, sometimes, of independent comorbidities in the form of rectal evacuation disorders described sporadically in patients with megacolon associated with MEN2B [5,53]. Colic transit time is generally prolonged. The intralumenal manometric study shows an increased compliance and a reduction of colic tonicity but with a diminished postprandial contractile reflex [53].

The evaluation of the resection piece is essential to confirm the definitive diagnosis of MI, which is true, retrospectively. In contrast to the preservation of submucosal ganglion cells on rectal biopsies, there may be significant histological changes in the deep layers (observed especially on resection specimens), such as decreased Cajal interstitial cell counts [54,55], decreased myenteric ganglia [56], decreased density of neural enteral structures [57] and/or smooth muscle hypertrophy [58]. These histological changes may precede the installation of the clinical picture as, moreover, these histopathological aspects can be noticed on the unenlarged colon segments [56].

As a conclusion (“stage”), it can be stated that if the suspicion of idiopathic megacolon can be raised from the first part of megacolon case management, the diagnosis of idiopathic megacolon can only be issued postoperatively by a histological evaluation of the resection piece. Obtaining detailed, useful, endoscopic histological data in the pre-surgical stage is almost impossible. On the other hand, this aspect is also the basis of the unspecified incidence of the disease (many of the cases with megacolon are strictly managed by non-surgical treatment).

A particular situation is that of patients who present urgently for a surgical complication with neglected diagnosis, and in whom the transit disorders require, on the one hand, a surgical intervention (often with emergency character). In these patients, the possibility of investigating the colon is limited precisely by the acute alteration of transit. Moreover, in our experience, most of these cases may be unknown with megacolon, which makes management even more difficult. In such a situation, from a technical point of view, performing barium enemas or colonoscopies from the diagnostic arsenal becomes illusory and, consequently, the only practical way available remains computed tomography (Figure 4) and simple abdominal radiography. Given the context-obstructive syndrome-these investigations are difficult to interpret diagnosis. The suspicion of idiopathic megacolon will, in these situations, be documented by the anatomical-clinical aspect of the intraoperative lesions and by the histopathological result of the resection piece.

## 5. Complications

The literature review presents a small number of communications in this regard, most being presentations of particular cases or extremely limited groups as the number of patients (of the order of tens). We found interesting, due to the secondary lesional extent of IM, a case of obstruction of the left iliac vein and left ureter at the pelvic opening, by compression determined by the impressive volume given the presence of the megacolon. Surgical treatment was welcome, with restitutio ad integrum for the described lesions [59,60].

Death is rarely described in cases with megacolon, most often due to perforation, the installation of peritonitis with severe sepsis or, more rarely, by hydro electrolytic disorders [61]. Ischemic phenomena in the dilated colonic segment with secondary shock and MSOF [62], compressive phenomena in the inferior vena cava [63], and bladder compression have also been reported.

By far, one of the most significant complications in IM in terms of frequency is that of an intestinal occlusion, either by impacting phachalomas or by volvulus [64]. There is an association of acquired megacolon with neuropsychiatric disorders, such as schizophrenia and mental retardation, as well as with organic disorders of the nervous system such as epilepsy and/or stroke [64]. The finding is a statistical conclusion without documenting a common etiopathogenic support. Moreover, complications occur more frequently in those with psychiatric disorders, and in this direction, we can speculate the delay in establishing the diagnosis and lack of rigor in monitoring, explained, at least in part, by the difficulties of cooperation and collaboration with the patient [65]. Here are also invoked the side effects of the use of psychotropic medication, which either favors by collaboration with etiological factors the evolution and installation of an IM or, as another possibility, “whips” the already established disease.

Even apart from actual complications, the consequences of the presence of the megacolon, through the described transit disorders, as well as the quasi-permanent abdominal discomfort lead to a significant degree of patient incapacity, difficult to quantify in terms of quality of life and little debated in the literature.

## 6. Therapeutic Management

The therapeutic decision involves an association of multiple competencies and specialties. Usually in this team are present the gastroenterologist, who assists these patients for a long time, both by evaluation and non-surgical treatment, the radiologist who monitors colorectal morphological changes, possibly complications, the surgeon especially in the therapeutic solution of complications or in case of failure of drug treatment, as well as other related specialties, such as a dietitian, psychologist, etc. Despite the unspecified etiology, patients with megacolon have excessive laxity, hypomotility, and rectal sensory dysfunction when evaluating anorectal function, leading to difficult digestive transit [66,67,68,69,70].

The first therapeutic option is conservative treatment, as most patients can be managed non-surgically [67]. However, drug treatment is ineffective in controlling symptoms in 50–70% of cases, and it can be difficult to tolerate, especially in the context in which it needs to be administered throughout life to avoid recurrence of symptoms [67,71,72,73]. This aspect becomes even more obvious in the case of the association with neuro-psychiatric disorders, where the patient’s compliance and cooperation is formally deficient. Moreover, drug treatment does not improve changes in the caliber of the colon even after years of sustained treatment, thus preserving the anatomical-clinical substrate of the symptoms [74]. Consequently, many patients resort to surgical treatment when conservative treatment proves ineffective or poorly tolerated [75]. The goal is to obtain a quality of life and is not to be neglected, this aspect having an important degree of subjectivism, especially in a patient with chronic suffering (such as the patient with idiopathic megacolon), where the sensitivity threshold is modified and difficult to quantify. Consultation and psychological support is therefore logical and mandatory. All the more so, as the long-term results of the surgical treatment do not guarantee the absence of the recurrence of the symptoms, being able to resort in certain situations to mutilating techniques such as colostomy (possibly definitive).

**Figure 8:**
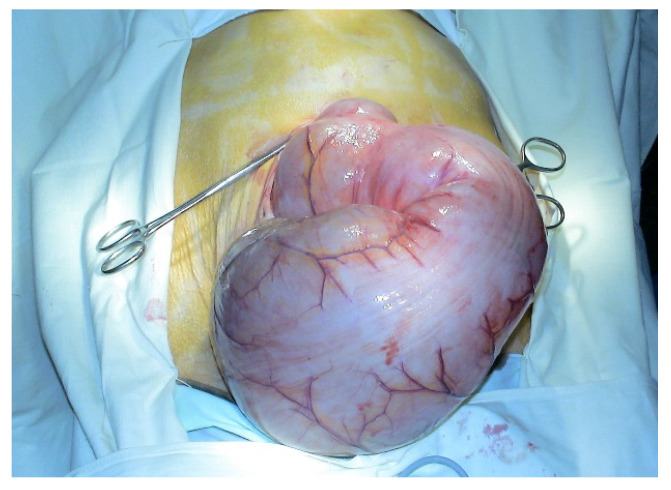
Intraoperative picture. Idiopathic megacolon, spectacular enlarged colon.

**Figure 9:**
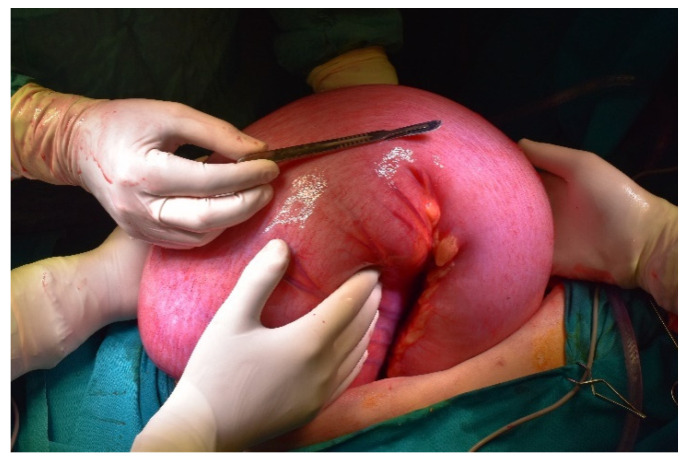
Intraoperative picture. Assessing the size of the colon.

On the other hand, some patients become surgical cases due to complications of the megacolon, such as obstructive/sub-obstructive syndromes (Figure 8 and Figure 9), for example by volvulation or, less frequently, colonic perforation [76,77].

However, it is necessary to synthesize some principles of surgical approach.

**Figure 10:**
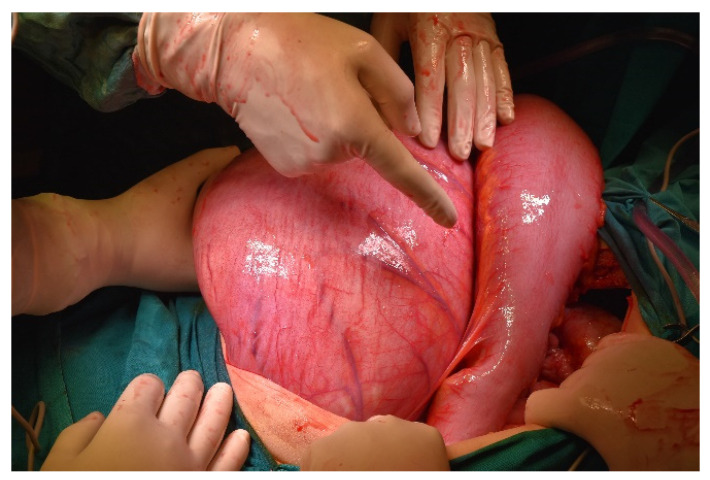
Intraoperative picture. Intrabdominal adhesions.

**Figure 11:**
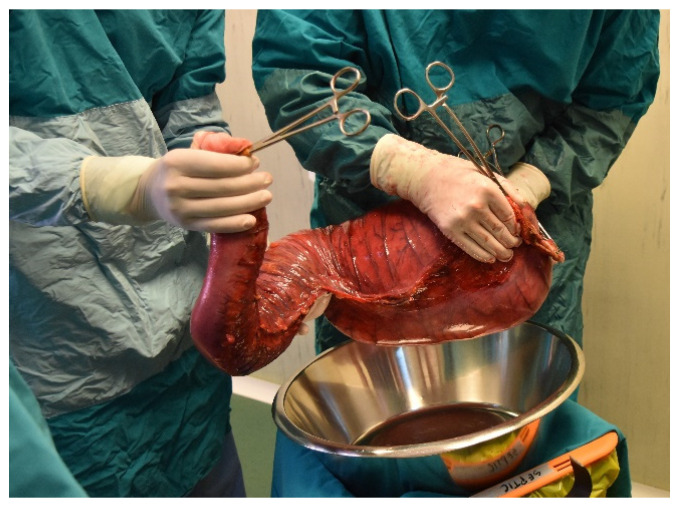
Intraoperative picture. Colon resection specimen.

In patients with idiopathic megacolon without rectal involvement, subtotal colectomy with ileorectal anastomosis is the technique of choice, because limited, segmental resections describe a higher rate of incidence of postoperative constipation (Figure 10 and Figure 11). Patients need to be advised that the technique is not innocuous, has a 20% morbidity rate (and even mortality) most often in connection with the onset of occlusive syndromes.

In the case of a simultaneous involvement of the colon and rectum, total proctocolectomy with ileo-anal anastomosis with ileal reservoir is recommended. The success rate is 70–80% with the inconvenience of a complex, pretentious technique, with the risk of improper functioning of the ileal reservoir, manifested by diarrhea or incontinence (especially at night).

In patients with distal dilatation (mega-rectum/sigmoid), the options are reduced between vertical reduction rectoplasty and rectal resection with coloanal anastomosis (with good results of about 70–80%). Logically, the first technique stated should be the first therapeutic option because it formally involves less surgical aggression, but the technique is overshadowed by the limited number of patients thus operated (implicitly with insufficiently evaluated long-term outcomes). Vertical reductional rectoplasty involves an anterior longitudinal incision of the dilated rectum, with resection of the anterior portion, leading to a significant reduction in rectal capacity [78]. No mortality with minimal early postoperative morbidity was recorded [78]. However, certainly, the hesitation to use this technique (based on the small number of operated cases) is due to the preference of surgical teams for a resection technique with colo-anal anastomosis due to the familiarity with this type of approach (technique, specifically modulated, it is much more commonly used in the surgical world for various colorectal pathologies).

The Duhamel technique with trans-anal descent is not recommended due to inconsistent results and high morbidity, often requiring additional surgical techniques for persistent constipation or complications [66].

Not to be overlooked, simple colostomy (or, as the case may be, ileostomy) can bring the same benefits while avoiding the risks specific to complex surgical techniques. Moreover, it is the only solution when other techniques fail. When opting for this type of approach, one must consider its positioning upstream of the dilated area and the lack of effectiveness on the symptoms of bloating or abdominal pain caused by dilated (and “abandoned” surgical segmental colorectal).

The pelvic-perineal surgical techniques are based on the hypothesis of a dysfunction of the ano-perineal and pelvic muscles with consequences on the colorectal evacuation and consequent dilatation upstream. Internal sphincterotomy has been little studied, describing favorable results in 1 of 3 cases when it was performed as a complementary intervention to another technique (resection) and in 2 out of 5 patients when it was used as a single intervention [79,80]. Kamm and colleagues proposed lateral disjunction of the puborectal fibers in the anal lifts, but performed in a limited number of patients and with more than controversial results [81].

Evaluation of data from the literature on the results of surgery for idiopathic megacolon should be done with caution. In addition, the comparison of the results of different studies is difficult due to major differences in the manner of performance (inclusion of cases, low statistical significance, questionable documentation of the lack of etiology of colorectal dilation, mandatory criteria for diagnosis of idiopathic megacolon, major differences in data, functional results and morbidity, etc.). To all this is obviously added the small number of patients included, justified by the low index of the incidence of the disease. In particular, there is a lack of data on long-term outcomes, obviously simple to justify by the slow evolution and varied addressability of the patient in various medical services [66].

## 7. Discussion

Idiopathic megacolon is a rare condition that equally affects both sexes with an unspecified etiology, which for decades has been a subject of controversy both etiologically and therapeutically. The most accepted hypothesis is the presence of changes in the intramural tendon network, in connection with collagen metabolism, most likely genetically determined, but in which environmental factors cannot be omitted. The very terminology for defining the disease is not unitary in the literature, using either the term “idiopathic megacolon” or “acquired megacolon”, in an attempt to suggest the absence of a precise etiology for the pathogenic substrate of the disease which, moreover, is relatively well documented. In this direction, we wonder if a more appropriate term would not be “acquired idiopathic megacolon”, to emphasize more clearly the anatomical-clinical substrate of the disease, whose pathogenesis is quite clear but without an etiology have an obvious trigger. The limited number of cases makes it difficult to unequivocally support this theory, as well as the other hypotheses promoted by studies focused on this topic. On the other hand, most of the histological changes proposed as a diagnostic basis are difficult to highlight on routine histopathological examinations, being “pretentious” and can be omitted in current practice. Moreover, currently and practically, if the diagnosis of megacolon is made relatively easily by imaging the colorectal dilatation (which is associated with consecutive clinical aspects), the formulation of the diagnosis of idiopathic megacolon is based almost exclusively on a principle of exclusion, after evaluating the absence of known causes that may lead to the installation of these anatomical-clinical changes (intramural aganglionosis, distal obstructions, intoxications, etc.). This has a direct consequence on treatment tactics. The absence of a certain etiology makes it impossible to develop an etiopathogenic therapy (the ideal form of treatment), all current treatment protocols being palliative therapies, aimed at improving the clinical picture (especially digestive transit disorders).

The interest in the condition from a surgical perspective is obvious. A large proportion of patients end up receiving surgical treatment after a period of treatment and monitoring in gastroenterology services. Regarding the surgical techniques of interest for this pathology, they address the anatomical-clinical epiphenomenon of the disease, namely the presence of the megacolon. Resection interventions are the most used due to their efficiency, but also in terms of the preference of surgical teams for this type of approach, in the sense of the learning curve already traveled during other pathologies, much more common and requiring different resection techniques (specifically modulated depending on the situation). “Specific” interventions addressed to the idiopathic megacolon, such as vertical reductional resection, require, above all, the documentation of efficiency, an aspect that is missing in the literature. On the other hand, the long-term efficacy of any form of treatment for idiopathic megacolon is not documented and communicated. However, observationally, it can be stated that most patients benefit from a permanently adapted protocol, in parallel with continuous monitoring, including drug treatment initially associated with hygiene and diet, followed by surgical treatment, especially resection techniques.

## 8. Conclusions

It is clear that the surgical management of idiopathic megacolon should be reserved for patients with symptoms refractory to drug treatment, with significant impairment of quality of life, with intolerance to non-surgical treatment. The management of the case must be done in a multidisciplinary team, with a good evaluation of the patient from a clinical, paraclinical and psychological point of view. This management is the sole responsibility of the surgeon for emergencies such as occlusion or perforation in the IM. Currently, no surgical technique is 100% effective, with the patient’s approval that surgery often only improves the clinical picture, and failure often involves opting for permanent colostomy.

## Figures and Tables

**Table 1:** Various pathological and non-pathological conditions that associate constipation as a clinically important aspect.

Functional Causes	Dietary Factors (Low Residue) Motility Disturbance (Slow Transit Time, Irritable Bowel Syndrome) Sedentary Lifestyle
Degeneration of enteric neurons	Chagas disease
Structural abnormalities	Anorectal disorders (anal or perianal fissures, thrombosed hemorrhoids) Colonic strictures (diverticulosis, ischemia, radiation therapy) Colonic tumor and obstruction (adenocarcinoma)
Smooth muscle and connective tissue disorders	Scleroderma, amyloidosis Toxic megacolon—inflammatory bowel disease (ulcerative colitis, Crohn disease, Cytomegalovirus, Salmonella, medications, etc)
Psychogenic conditions	Anxiety, depression, somatization
Neurogenic conditions	Cerebrovascular events, sclerosis, Parkinson’s disease, Hirschsprung’s disease, spinal cord tumors
Endocrine and metabolic conditions	Hypo- and hyperparathyroidism, diabetes mellitus, hypo- and hypercalcemia, uremia
Drugs	Antidepressants, narcotics, psychotropics, sympathomimetics, anticholinergics, diuretics, antacids, non-steroidal anti-inflammatory drugs, calcium channel blockers, etc.

## Abbreviations

IM	Idiopathic megacolon
HP	Histopathology
IHC	Immunohistochemistry
CT	Computerized tomography
MEN 2B	Multiple Endocrine Neoplasia, Type 2B
MSOF	Multiple systems organ failure
